# Patient‐Informed Biomechanical Modeling Reveals Mechanical Mechanism of Brain Damage in Idiopathic Normal Pressure Hydrocephalus

**DOI:** 10.1002/smmd.70060

**Published:** 2026-10-30

**Authors:** Vahid Darvishi, Martina Del Giovane, Michael C. B. David, Balvinder K. Dhillon, Magdalena A. Kolanko, Anastasia Gontsarova, Paresh A. Malhotra, Christopher Carswell, David J. Sharp, Mazdak Ghajari

**Affiliations:** ^1^ HEAD Lab, Dyson School of Design Engineering Imperial College London London UK; ^2^ Department of Brain Sciences Imperial College London London UK; ^3^ UK Dementia Research Institute Care Research and Technology Centre Imperial College London and the University of Surrey London UK; ^4^ Department of Neurology Imperial College Healthcare NHS Trust London UK

**Keywords:** brain biomechanics, computational biology, finite element modeling, normal pressure hydrocephalus, white matter

## Abstract

Idiopathic normal pressure hydrocephalus (iNPH) is a globally growing neurological disorder in older adults that is radiologically characterized by enlargement of ventricles. However, it remains unknown whether ventricular enlargement can produce biomechanical loading large enough to drive brain morphological changes and tissue damage. Here, we develop an anatomically detailed biomechanical model of aging human brain and apply ventricular enlargement using a three‐dimensional displacement field derived from MRI of iNPH patients and age‐matched controls. The model accurately reproduces radiological markers of iNPH, including Evans index, callosal angle, and high‐convexity sulcal narrowing. It further predicts large mechanical strains in the periventricular white matter, particularly within the corona radiata, corpus callosum, and anterior thalamic radiations, tracts consistently implicated in iNPH imaging abnormalities. These findings provide strong evidence that ventricular enlargement induces mechanical strain that contributes to iNPH brain abnormalities, which can potentially be reversed by reducing strain following shunting surgery. The biomechanical brain model forms the foundation of a predictive digital platform and future “digital twin” technology to support diagnosis, patient stratification, and treatment planning in iNPH.

## Key Points

- We develop an anatomically detailed biomechanical model of the brain, incorporating sulci, septum pellucidum and all four ventricles.
- A novel data‐driven loading approach is introduced which uses 3D displacement fields from finite element‐based registration of healthy and iNPH patient MRI, replacing the arbitrary pressure gradients of previous models.
- The model predictions closely match established radiological markers measured in iNPH patients, including Evans index, callosal angle and high‐convexity sulcal narrowing.
- Ventricular enlargement generates large mechanical strains concentrated in periventricular white matter, providing a biomechanical explanation for the structural abnormalities observed in iNPH.

## Graphical abstract

A patient‐informed biomechanical model of the brain reproduces key radiological markers of idiopathic normal pressure hydrocephalus during simulated ventricular enlargement. The model predicts large mechanical strains in the periventricular white matter, providing a biomechanical explanation for structural abnormalities observed in iNPH.

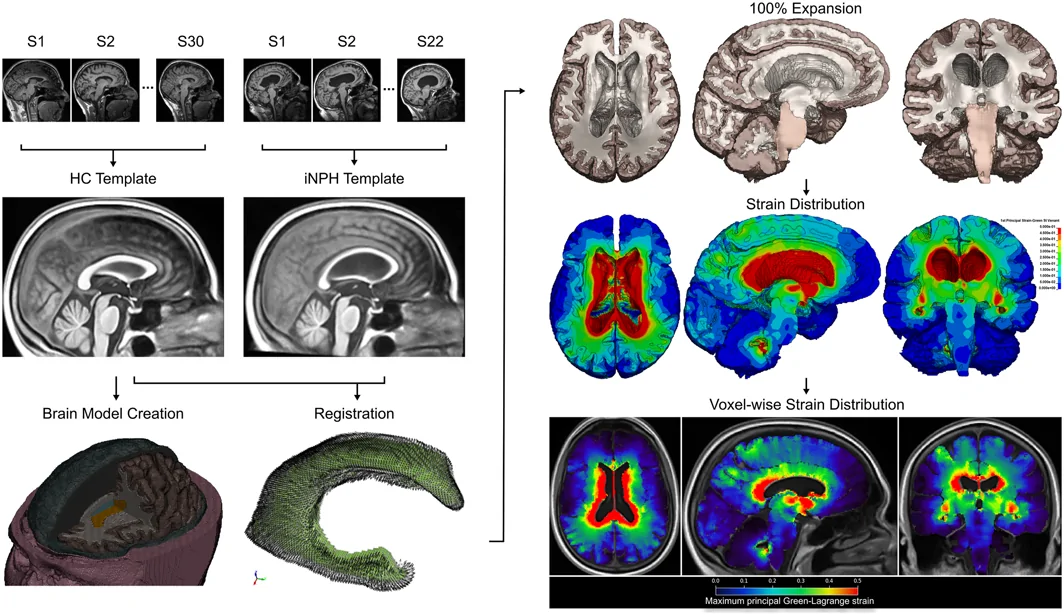

## 1 Introduction

Idiopathic normal pressure hydrocephalus (iNPH), first introduced by Hakim and Adams in 1964, is a neurological disorder characterized by abnormal enlargement of the ventricles and symptoms including gait disturbance, urinary incontinence, and cognitive impairment, also known as Hakim's triad [1, 2, 3, 4, 5, 6]. iNPH primarily affects individuals over age 60, with a prevalence of 1.5%–2.5% in those over 65 and up to 6% in those over 80 [7, 8]. With the global population aged 65 and older projected to double by 2050, the burden of iNPH will likely increase significantly in the coming decades [9].

iNPH is often misdiagnosed as Alzheimer's disease (AD) due to their overlapping symptoms and radiological features [10, 11]. However, unlike AD, iNPH can be reversed in some cases [12]. Cerebrospinal fluid (CSF) shunting improves symptoms in a proportion of patients [13, 14, 15, 16, 17], although recent evidence indicates that this benefit is partial and may not be sustained, and approximately 15% of all shunt procedures in the UK are performed for iNPH [18]. However, the reasons as to why a proportion of patients do not benefit from shunting remain unclear, likely due to limited understanding of the disease's underlying mechanisms [3, 4].

iNPH is likely to have a biomechanical nature, distinguishing it from hydrocephalus ex vacuo, where ventricular enlargement occurs passively to fill the space left by shrinking brain tissue, as commonly seen in AD. Studies have suggested that CSF accumulation within the ventricles applies pressure on the ventricular walls, contributing to their expansion and brain deformation [19, 20, 21, 22, 23]. In addition, early changes in the mechanical properties of brain tissue leading to secondary ventricular enlargement have also been suggested as another explanation for iNPH [24]. However, limitations in experimental and clinical methods for measuring biomechanical variables such as pressure gradients and brain deformation make it difficult to investigate the potential biomechanical mechanisms involved in iNPH [25].

Although the mechanisms underlying iNPH remain unclear, their effects consistently manifest as distinct macroscopic radiological features that form the basis of the clinical diagnosis of iNPH. Ventricular enlargement, the principal radiological sign of iNPH, is typically quantified using the Evans index (EI) and z‐Evans index (z‐EI), which measure the height and width of the ventricles, and callosal angle, which assesses the angle between the two lateral ventricles [26, 27]. Narrow CSF spaces at the high midline and high convexity areas are other radiological characteristics of the disease [22, 28, 29]. Although these features are routinely used in clinical practice, it remains unclear how ventricular enlargement leads to such characteristic morphologies and whether these macroscopic changes are associated with tissue damage, particularly white matter abnormalities.

Several studies have identified white matter abnormalities in periventricular regions in iNPH patients by using diffusion weighted imaging (DWI) [30, 31, 32, 33, 34, 35]. Axonal injury is thought to underlie the DWI abnormalities observed in white matter [4], representing microscopic hallmarks of iNPH. It has also been suggested that tracts located in periventricular areas are related to the clinical symptoms of iNPH. For instance, the corpus callosum, anterior thalamic radiations and corticospinal tract have been associated with gait disturbance [30, 36, 37, 38, 39], and the anterior thalamic radiations and cingulate cortex and its related tracts have been linked to urinary incontinence [40]. The spatial proximity of these tracts to regions of ventricular expansion suggests that mechanical factors may contribute to their abnormalities. In parallel, mechanical strain, which is a measure of local brain tissue deformation, has been used as a marker of tissue damage in traumatic brain injury, where regional strain distribution has been shown to predict the location of brain tissue damage and explain symptoms [41, 42, 43, 44]. These findings support the possibility that mechanical loading may contribute to the white matter structural abnormality and symptoms of iNPH.

Computational models that incorporate the anatomy and biomechanical properties of different tissues hold promise in investigating the relationship between brain mechanical loading and structural abnormalities in iNPH. Biomechanical computational models of iNPH have been developed in recent years. While some models incorporate a realistic description of brain anatomy [45, 46, 47, 48, 49], most focus on predicting CSF velocity or pressure field, rather than brain tissue deformation. A few studies focused on predicting brain deformation and tissue damage indicators, such as strain distribution, but their models lack key anatomical features [47, 49, 50, 51, 52, 53]. For instance, none of the previous models have incorporated 3rd and 4th ventricles and their expansion during iNPH. They also have not incorporated the entire anatomy of lateral ventricles, particularly the inferior regions. Moreover, they have used MRI scans of young adults, thus not accounting for the differences between younger and older healthy brains, or they have used only one subject's scan. Another limitation of previous computational models of iNPH is the loading condition used to simulate ventricular expansion. They expand the ventricles by applying an inaccurate or arbitrary transmantle pressure gradient (the pressure difference between the cranial subarachnoid space and the lateral ventricles) on the boundary of the ventricles. However, this approach produces an unrealistic shape of expanded ventricles, leading to an inaccurate prediction of brain deformation [47]. As such, no computational models have been able to predict the key radiological features of iNPH, nor have they explained the characteristic white matter abnormalities seen in iNPH.

Here, we develop a new patient‐informed computational model of iNPH, which incorporates fine anatomical details of key brain regions, including all four ventricles and sulci. We also introduce a new loading condition derived from MRI scans of iNPH patients and age‐matched healthy controls to accurately capture the morphology of enlarged ventricles. We evaluated whether this novel biomechanical model can accurately predict key radiological markers of iNPH. In addition, motivated by the spatial correspondence between ventricular morphology and periventricular white matter abnormalities in iNPH patients, we investigated the validity of the model's mechanical strain predictions in identifying the white matter tracts often reported as abnormal in previous studies [54, 55]. This study can improve our understanding of the role of biomechanics in iNPH and establish the computational modeling framework as the foundation for a digital platform for improving iNPH diagnosis, patient stratification and planning of shunting interventions.

## 2 Results

### 2.1 The Anatomically Detailed Finite Element Model of Brain

The MRI scans of 30 healthy controls (HC) (16 males, mean age = 73.80 ± 5.71) were averaged to create a template. An in‐house pipeline was then used to convert the template into a finite element (FE) model of the brain [44, 56]. Anatomical features, including sulci, pia, falx and tentorium, were also generated using the pipeline (Figure 1a,b). Manual segmentation of the ventricles and septum pellucidum (SP) of the template resulted in an accurate representation of their fine anatomy (Figure 1c,d). This included the lower parts of the ventricles, which are missing in previous models [47, 49, 50, 51, 52]. Hyperelastic isotropic Ogden material models with constants extracted from experiments on human brain tissues were assigned to different anatomical regions to predict the nonlinear mechanical response of the tissues to loading [57, 58].

**FIGURE 1:**
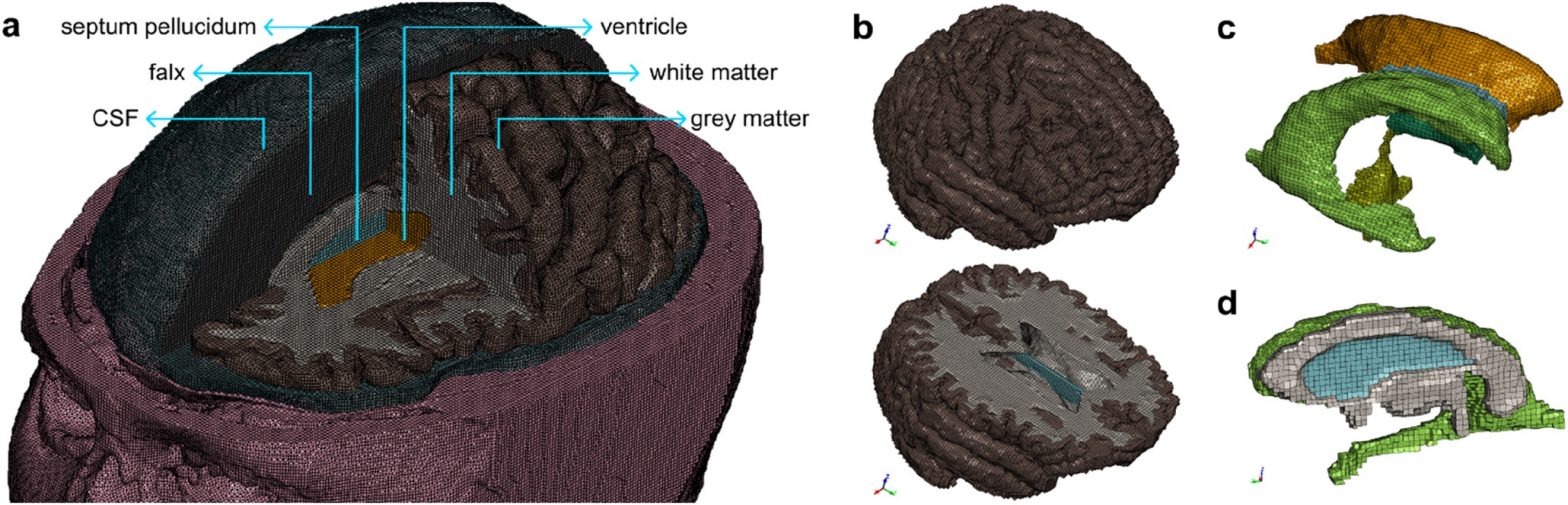
The anatomically accurate finite element model of the brain, representing healthy old adults. (a) Geometry of key brain regions after incorporation of anatomical features, including sulci, falx, septum pellucidum (SP), and pia mater, (b) Detailed representation of gyri and sulci, (c) Refined and anatomically accurate ventricular system used in this study and (d) Anatomy of SP shown in blue.

### 2.2 Patient‐Informed Ventricular Expansion

To reproduce ventricular expansion, a three‐dimensional displacement field was applied to the lining of the ventricles. This displacement field was obtained by mapping the ventricles of the HC template to the ventricles of an iNPH template, created from the MRI scans of 22 iNPH patients (14 males, mean age = 71.91 ± 7.06). Compared with linear and nonlinear image registration, the FE‐based registration provided superior alignment with iNPH ventricles (Figure 2). Using the node‐wise surface comparison, we found a 95th percentile Hausdorff distance (HD95) [59] of 2.2 mm for the FE‐based registration, versus 12.3 mm for the linear and 4.7 mm for the nonlinear image registrations. Hence, the FE‐based registration was used to determine the displacement field of the ventricular linings.

**FIGURE 2:**
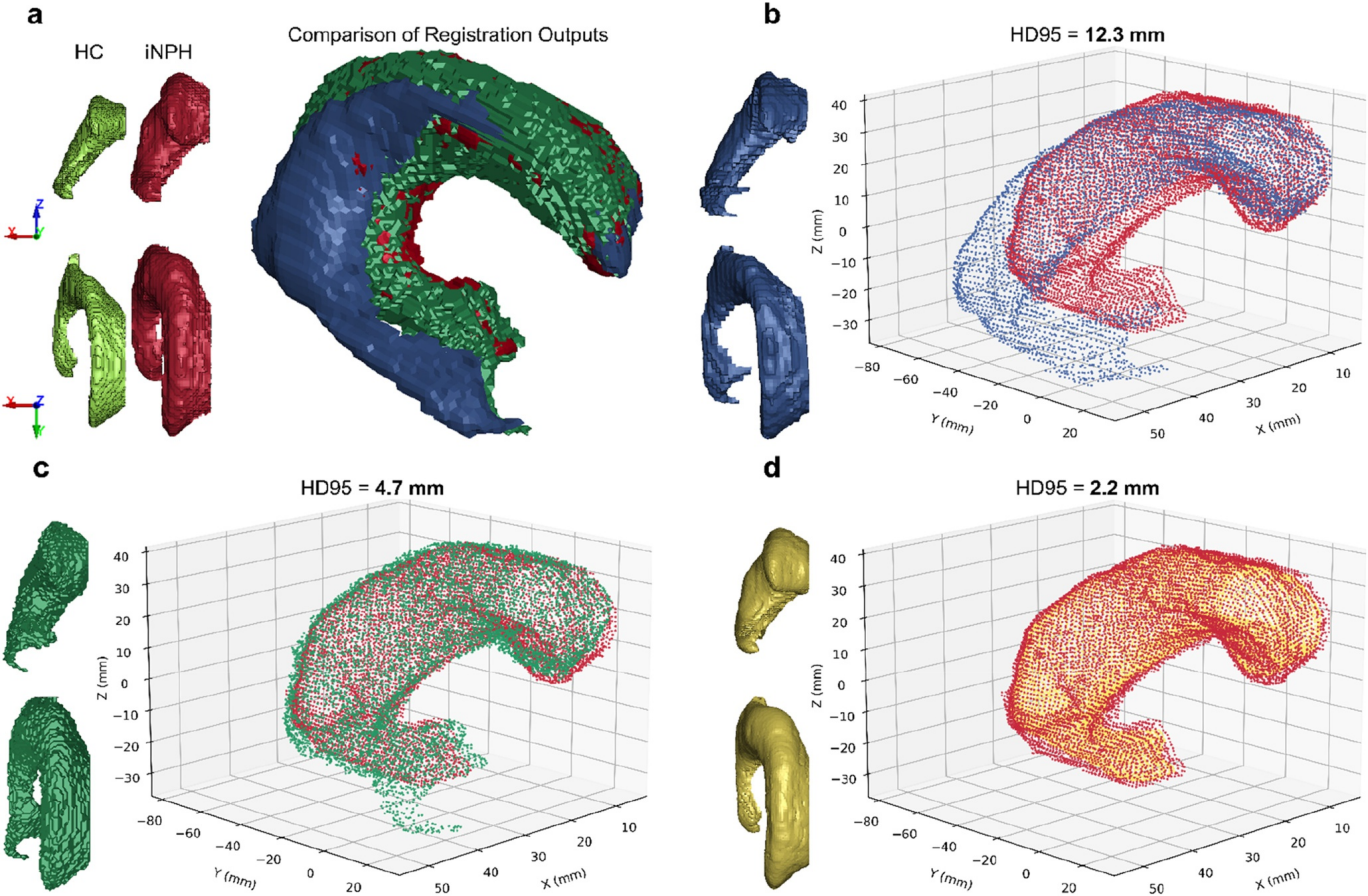
Different registration methods for aligning HC and iNPH ventricles. This figure shows the registration and shape analysis of the right lateral ventricle. (a) Anterior‐posterior and superior‐inferior view of the right ventricle of HC (green) and iNPH (red), along with an isometric view of the registered versions of the right ventricle. (b) FSL linear registration result (blue), evaluated against iNPH. (c) ANT nonlinear registration output (dark green), evaluated against iNPH. (d) FE‐based registration output (yellow), evaluated against iNPH.

The lining of the ventricles was made of approximately 21,000 finite element nodes. The displacement fields were converted into displacement vectors, which were used to prescribe the displacement of these nodes. Ventricular expansion was modeled by increasing the magnitude of these vectors simultaneously from zero to their maximum value (Figure 3).

**FIGURE 3:**
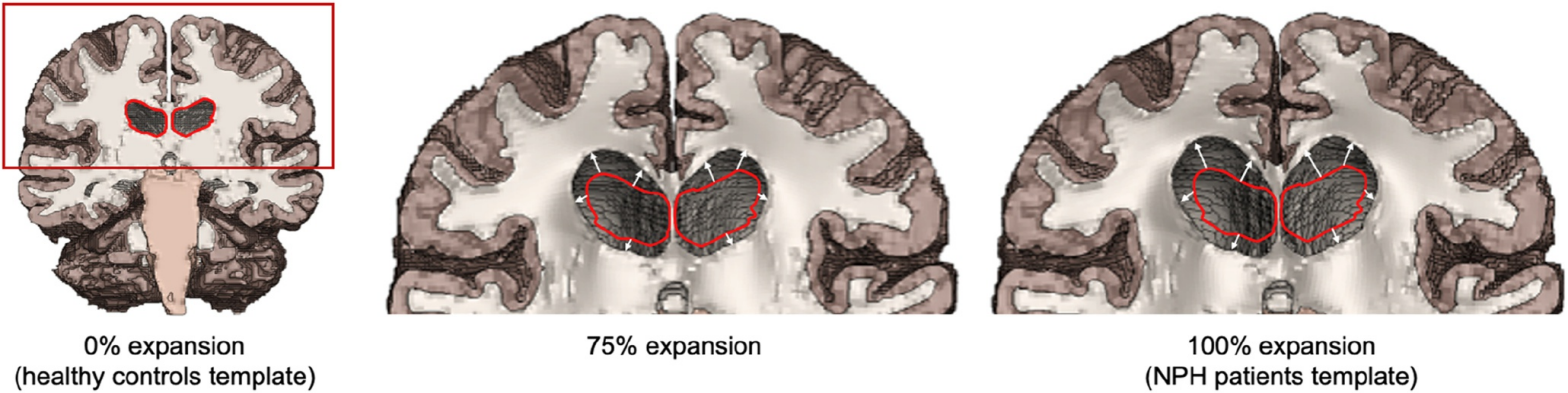
Simulation of ventricular expansion by using a displacement field. The red outlines show the lining of the ventricles in the HC template. The white arrows show the displacement vectors, determined from the FE‐based registration of the ventricular lining of HC to iNPH.

### 2.3 The Model Reproduces Ventricular Morphology Metrics

Evans index (EI) and z‐Evans index (z‐EI) are widely used radiological markers for iNPH. EI is defined as the ratio of the maximum distance between the frontal horns of the lateral ventricles to the maximum internal diameter of the cranium in the lateral direction (Figure 4a). Similarly, the z‐EI is defined as the ratio of the maximum z‐axial distance between the upper and lower parts of the frontal horn to the maximum internal diameter of the cranium in the *z* direction, measured at the posterior margin of the interventricular foramen (Figure 4b). These indices were measured in iNPH patients by a neuroradiologist and were also predicted by the brain model during ventricular expansion simulation (Figure 4c,d). The EI of the FE model increased linearly from 0.283 to 0.345 as the ventricles fully expanded, falling within the range measured in patients and differing by only 4.4% from the patients' median (Figure 4e). The z‐EI also increased linearly from 0.306 to 0.417, with a deviation of just 1.8% from the patients' median (Figure 4f).

**FIGURE 4:**
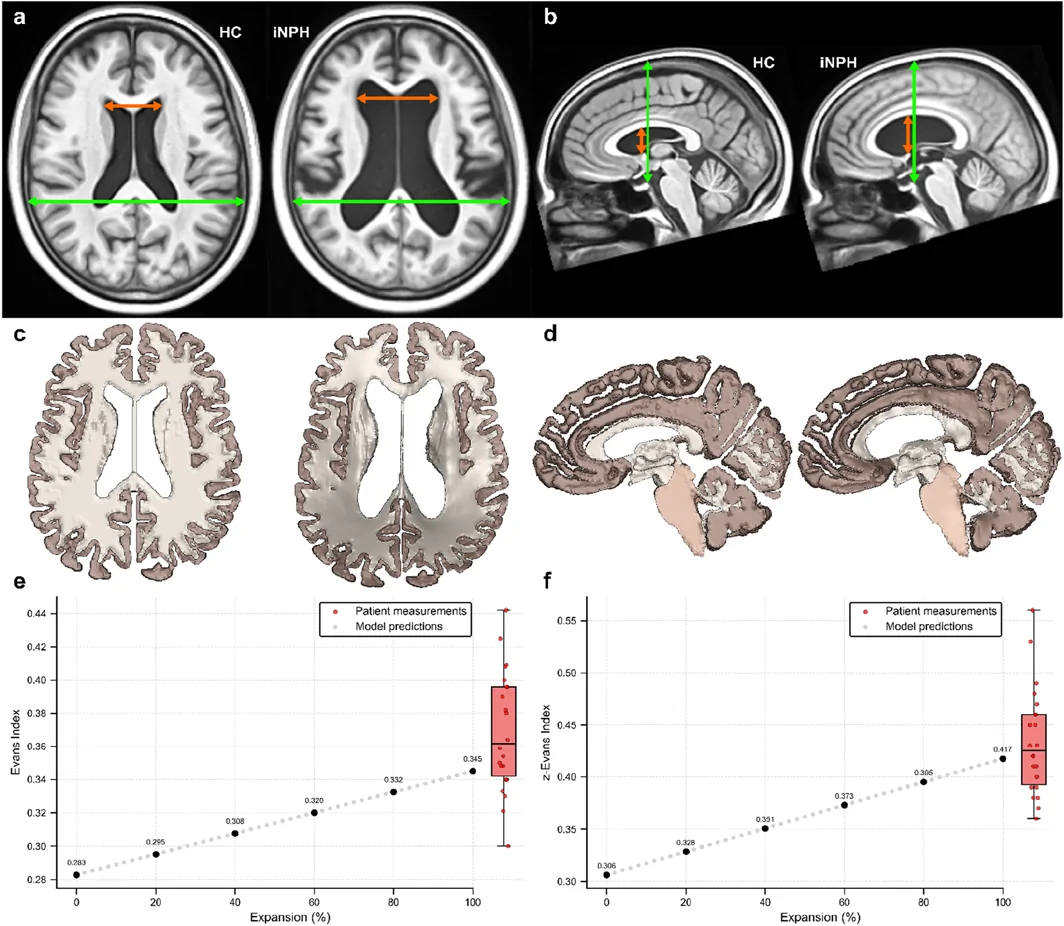
Comparison of EI and z‐EI between MRI templates and the brain model. (a) Definition of EI. (b) Definition of z‐EI. (c) Increase in the EI predicted by the model. (d) Increase in the z‐EI predicted by the model. (e) EI measurements show a close agreement between data extracted from iNPH patients' scans and model predictions. (f) z‐EI measurements show a close agreement between data extracted from iNPH patients' scans and model predictions.

The reduction in the callosal angle, the angle between the lateral ventricles, is another indicator of iNPH (Figure 5a) [60, 61, 62]. This angle is measured on the coronal plane perpendicular to the anterior commissure‐ posterior commissure line at the level of the posterior commissure. The FE model predicted a slightly nonlinear reduction in the callosal angle, decreasing from 116.6° before ventricular expansion to 71.4° at full expansion, corresponding to only a 0.7% relative error compared with the median callosal angle of iNPH patients (Figure 5b,c).

**FIGURE 5:**
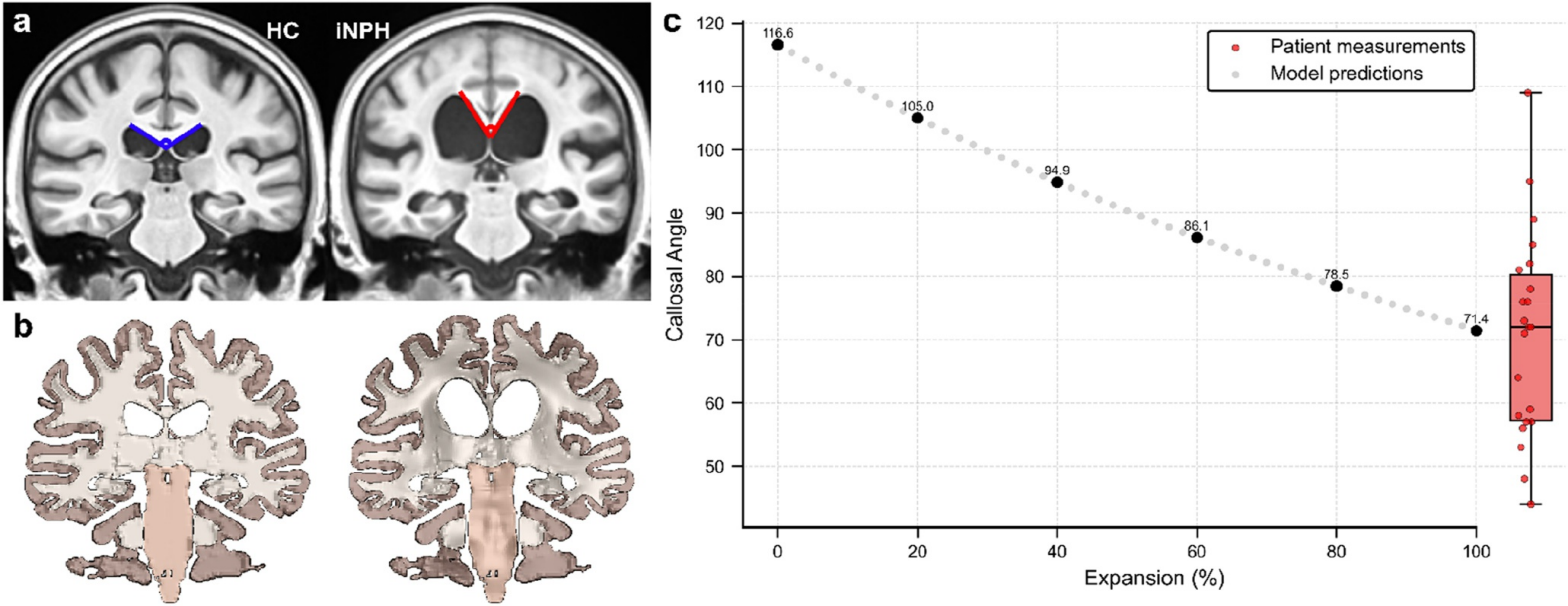
Callosal angle during expansion of ventricles in iNPH. (a) Definition of Callosal angle and its measurement in MRI templates. (b) Reduction of callosal angle predicted by the model. (c) Change in callosal angle over time during ventricular expansion predicted by the model, which is in a close agreement with measurements from iNPH MRI scans.

### 2.4 Narrowing of Interhemispheric Fissure and Sulci

We also examined broader brain morphological changes induced by ventricular expansion. A reduction in the CSF space at high midline and high convexity areas has been reported in iNPH patients, seen on MRI as narrowing of the interhemispheric fissure and superior cortical sulci [22, 28, 29]. Consistent with these observations, the brain model predicts a progressive decrease in the distance between the left and right hemispheres as the ventricles expand, with the greatest reduction in the posterior regions (Figure 6a). Likewise, the model predicts narrowing of sulci in high convexity areas, which is again more pronounced in the posterior regions (Figure 6b).

**FIGURE 6:**
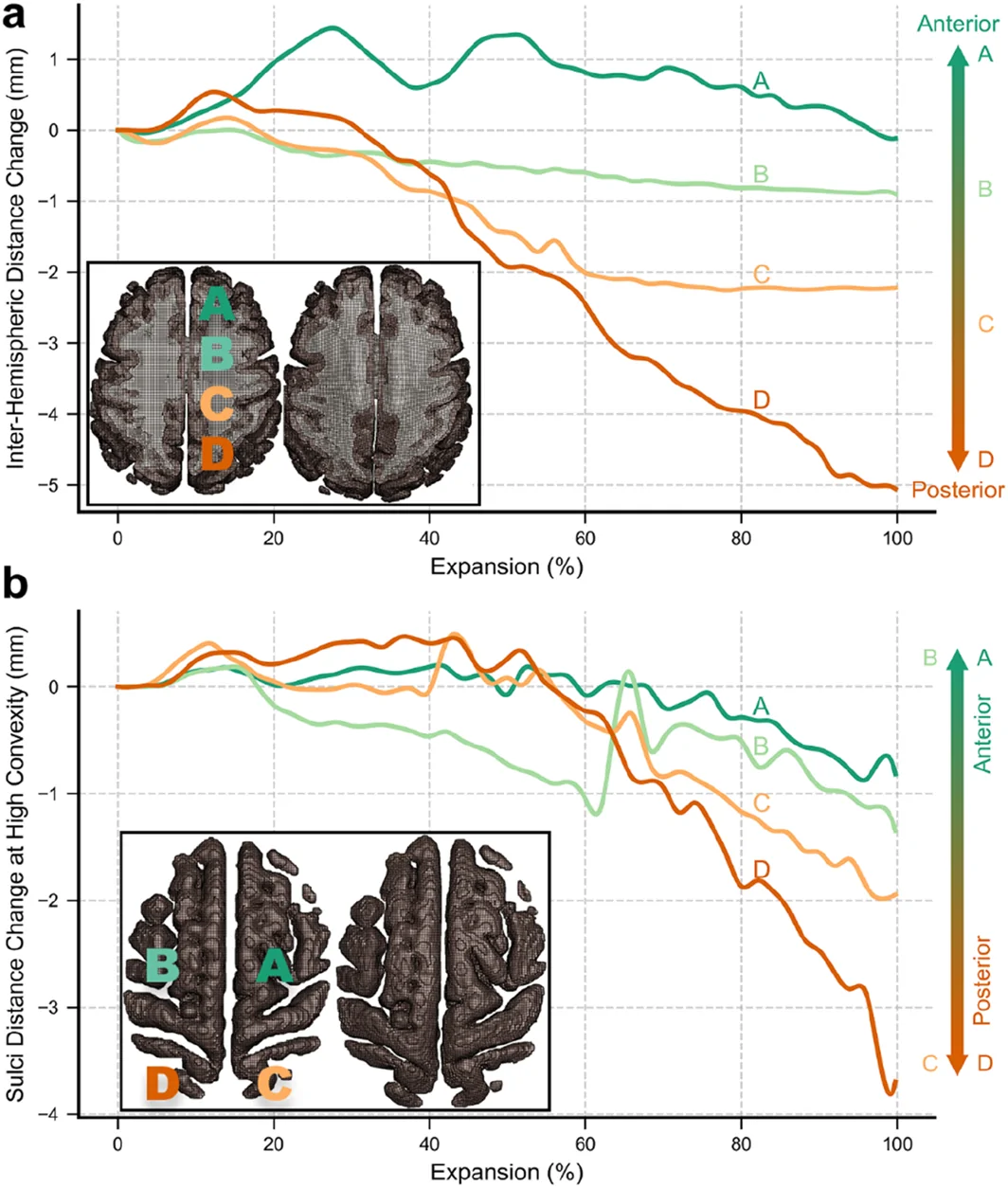
Predicting interhemispheric fissure and sulci narrowing at high convexity in iNPH. (a) Interhemispheric distance reduction at multiple locations during the ventricular expansion, with the greatest reduction in posterior locations. (b) Narrowing of the sulci at high convexity, which is more pronounced in posterior regions.

### 2.5 Reduction in Septum Pellucidum Thickness

The septum pellucidum (SP), the thin layer between the lateral ventricles, has been highlighted as a potential region of interest for brain‐related neurological conditions [63]. We observed a reduction in SP thickness when comparing the HC and iNPH MRI templates, with the reduction being more pronounced in the posterior region (Figure 7a). The brain model predicted that the SP thickness decreases nonlinearly as the ventricles expand, with greater thickness reduction in the posterior region of the SP (Figure 7b).

**FIGURE 7:**
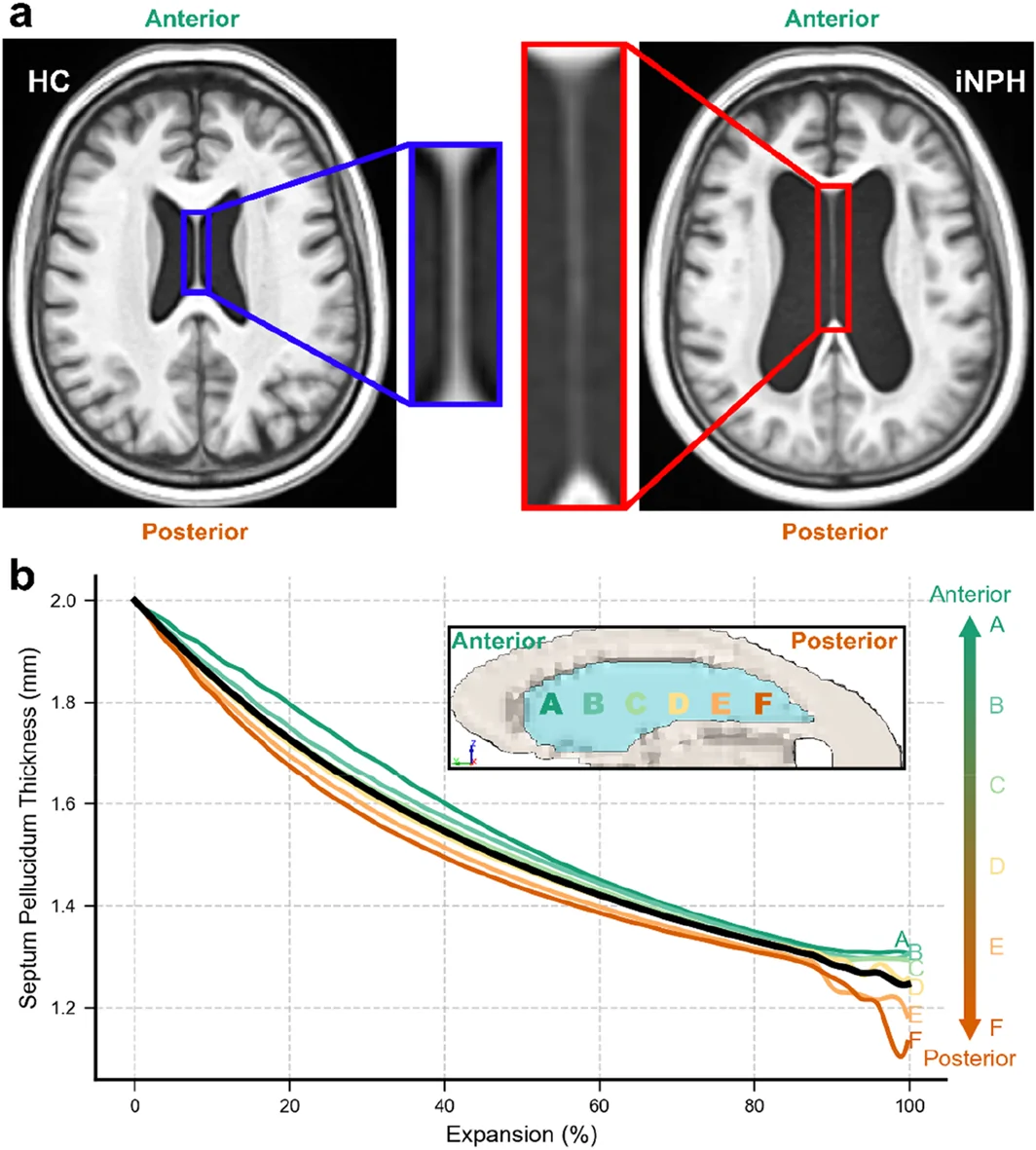
SP thickness reduction during ventricular expansion observed in MRI scans of patients and predicted by the brain model. (a) SP thickness in HC and iNPH MRI templates, showing reduction in iNPH SP thickness, which becomes more pronounced in the posterior regions. (b) The brain model predicts a continual reduction in SP thickness during iNPH progression, with reductions more pronounced in posterior regions. The black line represents the average reduction.

### 2.6 Large Strains in Areas of White Matter Abnormality

The distribution of maximum principal Green‐Lagrange strain across the brain, produced by ventricular expansion, was predicted by the brain model. The largest strains were concentrated in the periventricular regions and most notably in the septum pellucidum and corpus callosum (Figure 8a). To determine strain distributions in white matter tracts, the JHU ICBM‐DTI‐81 white matter tract atlas was used [64]. The 95th percentile strain in white matter tracts ranged from 0.15 to 0.98 (Figure 8b). The tracts with the largest 95th percentile strain were the posterior corona radiata, corpus callosum splenium and body, and anterior thalamic radiations. These tract‐level strains were verified to be independent of the ventricular expansion duration. Although the kinetic‐to‐internal energy ratio fell below the conventional 5% threshold from 50 ms onward, individual tracts required durations of 250 ms or longer before all 95th percentile strains fell within 5% of a 1000 ms reference. At the 500 ms duration used here, the largest deviation of any tract‐level strain was below 2.2% (Supporting Information S1: Figure S2).

**FIGURE 8:**
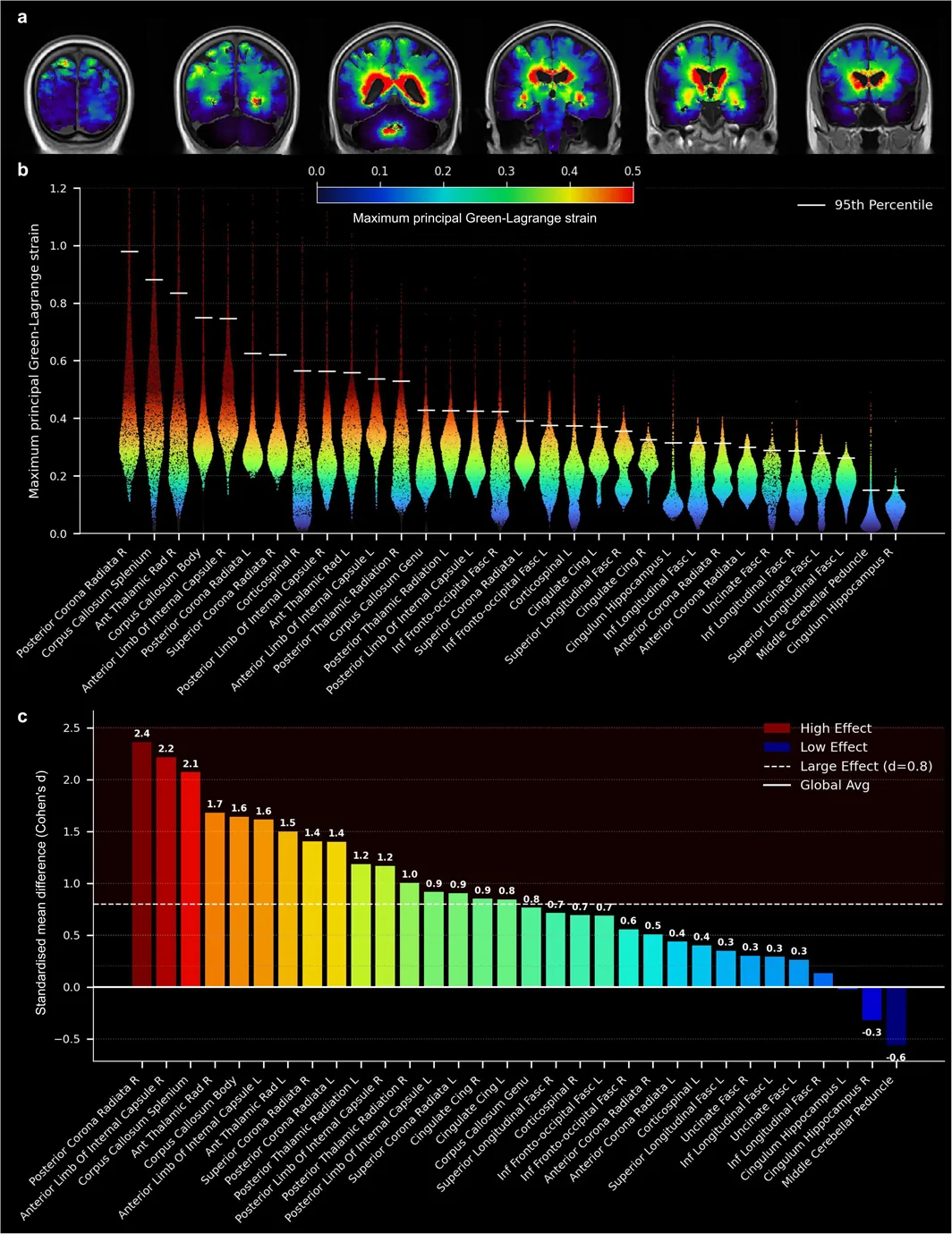
Maximum principal Green‐Lagrange strain distribution predicted by the iNPH brain model. (a) Maximum strain in different regions of the brain. (b) Strain distribution across white matter tracts, based on maximum principal strain analysis. (c) Standardized mean difference (Cohen's d) between the strain within each white matter tract and the whole‐brain strain distribution, using the pooled standard deviation of the two. Sixteen of the 32 tracts exceeded the large‐effect threshold of *d* = 0.8 (dashed line). The periventricular tracts experience the largest effects, with d up to 2.4.

To identify the white matter tracts experiencing significantly higher levels of strain compared to the whole brain average, the effect size (Cohen's d) was calculated. The Cohen's d is a measurement of the magnitude of the difference between the tract's strain and the global average, expressed relative to the overall variability in strain. The largest effect sizes were measured for the posterior corona radiata, anterior limb of the internal capsule, corpus callosum splenium and body, and the anterior thalamic radiations, with Cohen's d values of 2.4, 2.2, 2.1, 1.6, and 1.7, respectively (Figure 8c).

### 2.7 Applicability of the Framework to Patient‐Specific Modeling

To evaluate whether the framework can reproduce the ventricular anatomy of individual patients, the FE‐based registration was repeated for all 22 patients in the cohort. The ventricles of each patient were segmented first and used in place of the iNPH template as the target surface of the registration.

The HC ventricle was successfully expanded onto the ventricles of every patient. Registration accuracy was quantified with the same HD95 measure used for the template registration, so the values are directly comparable. The median HD95 was 1.12 mm (mean 1.47 ± 0.62 mm; range 0.93–2.98 mm). Every registration fell below 3 mm, and the majority were at or below the 2.2 mm obtained for the template registration (Figure 9a). Accuracy was maintained across the full range of ventricular enlargement in the cohort, and the most enlarged ventricles were among the most accurately registered. The registrations with the smallest and the largest HD95 are shown in Figure 9b,c. The registration results for all patients are shown in Supporting Information S1: Figure S4.

**FIGURE 9:**
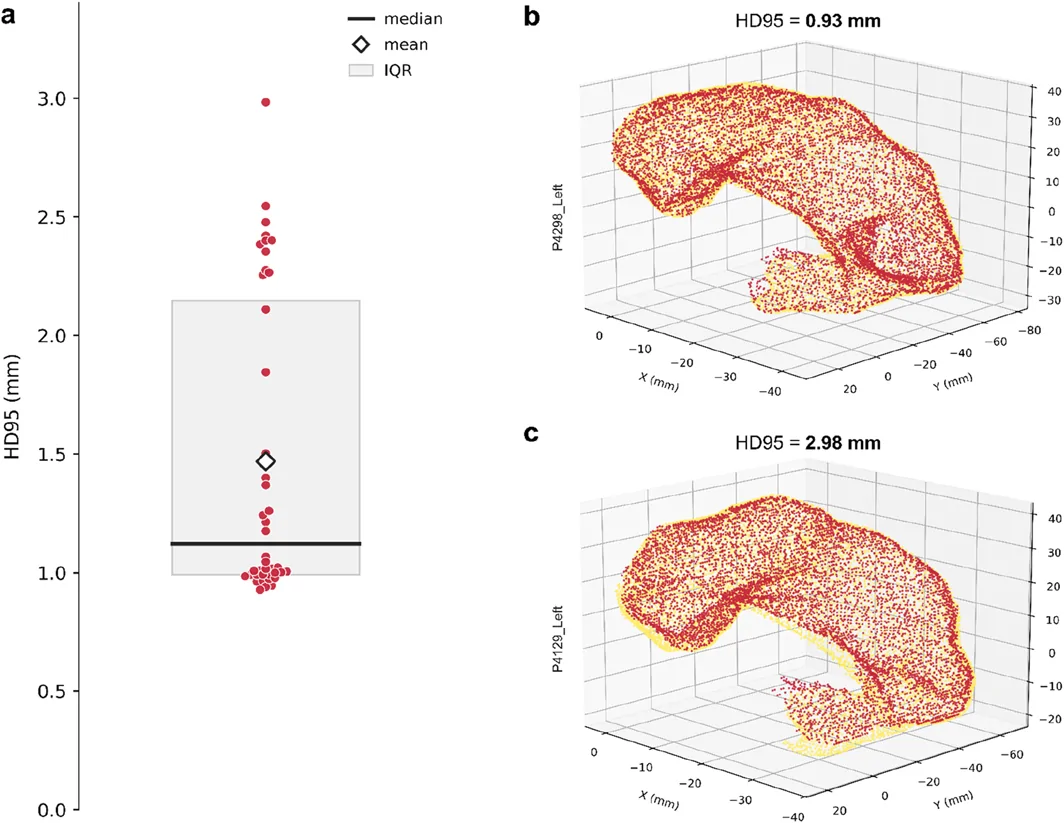
Applicability of the framework to individual patients. (a) Distribution of the HD95 between the registered HC mesh and each patient's segmented ventricle. The solid line and the diamond represent the median and the mean, respectively. Registered HC mesh (yellow) overlaid on the patient's segmented ventricle (red), for the most accurate registration ((b) HD95 = 0.93 mm) and the least accurate registration ((c) HD95 = 2.98 mm).

## 3 Discussion

We show that an anatomically detailed biomechanical model of the brain loaded by displacement of the ventricular lining, can accurately reproduce established radiological markers of the iNPH disease, including Evans index, z‐Evans index and callosal angle. Moreover, the model successfully predicts morphological changes seen in MRI of iNPH patients, including narrowing of high convexity sulci and interhemispheric distance reduction. In addition, the model predicts large mechanical strains in white matter tracts where abnormalities have been seen in diffusion imaging of iNPH patients. Together, these findings indicate that brain biomechanical loading resulting from enlargement of ventricles plays an important role in iNPH pathophysiology. They also validated model predictions, supporting the use of this model in future studies on understanding iNPH mechanisms and improving its diagnosis and treatment.

A significant advancement of the presented brain model, compared with previous models of iNPH [46, 47, 48, 49] (Table 1), is the incorporation of anatomical features that influence brain deformation during ventricular enlargement, such as sulci and falx. The falx may play a role in constraining adjacent areas such as the corpus callosum during ventricular expansion. In contrast, the sulci and CSF space at high convexity areas provide space for brain tissue movement as ventricles enlarge, thus reducing brain deformation and strain in these areas. In addition, an accurate definition of ventricular anatomy, including the lower parts of the lateral ventricles and 3rd and 4th ventricles, was incorporated in the model. This enabled the model to predict areas of elevated strain in the vicinity of ventricles as they enlarge (Figure 8a). This accurate and detailed prediction of neuroanatomical distribution of mechanical strain may help understand abnormalities observed in iNPH.

**TABLE 1:** Comparison of the present model with prior biomechanical models of iNPH and related hydrocephalus.

Study (Year) [Ref]	Dim.	Anatomical features	Ventricles	Template/Subject	Loading	Radiological validation	Tract‐level analysis
Taylor and Miller (2004) [51]	2D	Not reported	Lateral	Generic	Pressure	None	No
Clatz et al. (2007) [45]	3D	Not reported	Lateral	Single patient	Pressure	Ventricular surface distance versus post‐shunt‐closure	No
Dutta‐Roy et al. (2008) [47]	3D	Not reported	Lateral	Generic healthy adult	Pressure	Ventricular volume versus literature	No
Cheng and Bilston (2010) [49]	3D	SP	Lateral	Brain atlas	Pressure	None	No
Chen et al. (2013) [46]	3D	Falx and tentorium	Lateral	4 patients	Pressure	Ventricular surface versus same patient's later MRI	No
Lefever et al. (2013) [48]	3D	Not reported	Lateral	Single male	Pressure	Ventricle volume versus clinical average	No
Rogers et al. (2014) [53]	3D	Falx	Lateral	Single subject	Pressure	Ventricle volume versus clinical average for noncommunicating hydrocephalus	Corona radiata and internal capsule from a single seed
Kim et al. (2015a) [50]	2D	Sulci, falx, gray/white	Lateral	Single subject	Pressure	Qualitative comparison	No
Kim et al. (2015b) [52]	2D	Sulci, falx, gray/white	Lateral	Single subject	Pressure	Volumetric strain contours	No
Gholampour and Fatouraee (2021) [65]	3D	Not reported	Lateral + 3rd + 4th (as CSF paths)	11 hydrocephalus patients + 8 healthy subjects	Pressure	None (CSF flow focus)	No
Present study	3D	Sulci, SP, falx, pia, dura, tentorium	All four (incl. lower lateral)	Age‐appropriate template (*n* = 30 HC, mean age 73.8)	MRI‐derived 3D displacement field (FE‐based registration)	EI, z‐EI, callosal angle, sulcal/IHF narrowing, SP thinning	32 atlas‐defined tracts

Another key novelty of the modeling approach is the use of MRI data to displace the ventricular lining, thus allowing for an accurate reproduction of the morphology of expanded ventricles. Previous models have failed to predict the shape of the expanded iNPH ventricles because they used pressure gradients to derive ventricular expansion, leading to a uniform expansion across ventricular regions [46, 47, 48, 49, 51, 52] (Table 1). The registration of HC to iNPH ventricles provided around 21,000 unique displacement vectors for the nodes of ventricles' linings. This method allowed for the accurate prediction of nonuniform expansion of the ventricles, closely reproducing the heterogeneous ventricular enlargement seen in iNPH patients, with excellent predicted values for the established radiological metrics of iNPH. The model demonstrated higher predictive accuracy for the z‐EI and callosal angle compared to the EI, which is due to the sensitivity of these measurements to initial segmentation boundaries and the surface smoothing required for numerical stability in the FE models.

The model predicted additional morphological changes, including narrowing of sulci at the high convexity, narrowing of the interhemispheric fissure and thinning of the septum pellucidum (SP). These changes were most pronounced in the posterior regions. Tight high convexity and compressed midline subarachnoid spaces are morphological features that suggest iNPH [22, 28, 29]. The model prediction of these features further supports the role of mechanical loading in driving iNPH morphological changes. The thinning of the SP has not been widely reported as an imaging feature of iNPH, possibly due to its small size, which complicates its measurement on MRI. Nonetheless, SP has been implicated in other neurological disorders [63]. The model predicts a substantial average reduction of approximately 37% in SP thickness due to ventricular enlargement, suggesting that SP thickness may be a useful morphological feature in the iNPH context.

The model predicted large mechanical strains in the periventricular white matter tracts, with the posterior corona radiata, corpus callosum, anterior thalamic radiations and anterior limb of the internal capsule sustaining the highest 95th percentile strain values. This observation was further supported by the effect size analysis, which identified these tracts as experiencing significantly higher strain levels than the global brain average (Cohen's *d* > 1.4). This aligns with MRI studies of iNPH patients, which consistently report diffusion abnormalities in periventricular regions, with the corpus callosum being reported in several studies [30, 31, 34, 66]. For example, one study comparing diffusion imaging of iNPH and Alzheimer's disease patients reported significant reduction in fractional anisotropy, which indicates abnormality, in the corpus callosum [31]. Consistent with these observations, we predicted high levels of strain in the corpus callosum. The relationship between mechanical stretching of white matter tracts and subsequent axonal injury has been established in animal studies [42, 67, 68]. Our results further confirm the ability of the computational model in predicting brain abnormalities in iNPH.

While verifying the predictions, we found that the kinetic‐to‐internal energy ratio and whole‐brain strain converged considerably earlier than tract‐level strains. At an expansion duration of 50 ms, the kinetic‐to‐internal energy ratio was 2.5% and the whole‐brain 95th percentile strain deviated by only 0.37% from the reference; however, the most‐affected tract still deviated by 7.2%, and 9 of the 32 tracts remained outside 2%. Because the whole‐brain statistic pools approximately 1 million voxels, opposing regional differences average out. This shows that a global measure can indicate convergence while regional estimates are not converged. Studies reporting regional or tract‐level strain should therefore verify convergence at the level at which the results are interpreted, rather than relying on global energy or whole‐brain strain alone (Supporting Information S1: Figure S2).

The biomechanical model predicted the highest strains in the corona radiata, corpus callosum and anterior thalamic radiations. These tracts have been most consistently associated with gait disturbance, an early and common symptom in iNPH [35, 36, 69]. This unique pattern of strain distribution may provide a biomechanical explanation for the clinical progression of iNPH symptoms. This suggests that the early appearance of gait disturbance could be a consequence of early and continued mechanical stretching of tracts associated with gait. Animal studies have shown that stretching white matter tracts beyond a threshold can initiate functional deficits, and continued deformation can result in irreversible axonal damage [68, 70, 71]. However, it is important to note that strain thresholds derived from high loading rate traumatic brain injury (TBI) models may not be directly used in iNPH. During the slow, progressive deformation characteristic of iNPH, axons may undergo biological accommodation and lengthening [72, 73], which likely increases the strain required to induce irreversible damage compared to acute impact scenarios. Therefore, our model utilizes strain primarily as a relative indicator of regional mechanical loading of brain tissue. Our model predicts monotonically increasing strains in all tracts as ventricles expand, meaning that eventually more tracts can reach a threshold of irreversible damage. This raises the possibility that interventions aimed at reducing ventricular expansion may not be effective if the tracts are permanently damaged, a potential reason for not responding to shunting in some patients.

This study highlights the key role of biomechanics in iNPH. While the brain model is purely biomechanical and does not account for potential biological or genetic factors, such as pre‐existing neurodegeneration or tissue softening prior to ventricular enlargement, the strong agreement between model predictions and common morphological and structural indicators of iNPH suggests that mechanical forces play an important role in deriving the observed abnormalities. It also implies that ventricular enlargement imposes mechanical loading on brain tissue, rather than being solely a consequence of early tissue softening. Clinical interventions that reduce CSF volume and pressure, such as shunting procedures or CSF‐suppressing pharmacologic treatments, have been shown to alleviate both imaging markers of white matter damage and patient symptoms [74, 75]. This therapeutic effect further supports the role of biomechanics in iNPH, where excessive CSF accumulation increases pressure at the ventricular surface, leading to tissue deformation and damage, which can be partially reversed when the mechanical loading is reduced.

Several material formulations have been used to model brain tissue, including hyperelastic, viscoelastic, poroelastic and biphasic models [47, 52, 76, 77], but there is no consensus that a poroelastic or biphasic formulation is required for chronic quasi‐static loading for example in iNPH. Since we focused on tissue deformation and strain distribution rather than fluid transfer, we used a hyperelastic Ogden model with prescribed displacement boundary conditions at the ventricular lining. The model also uses simplified boundary conditions at the pia–skull interface, a simplification common in brain finite element modeling. Mechanisms such as *trans*‐ependymal CSF flow and interstitial edema have also been reported in iNPH patients but were not included in the current model explicitly [34, 78, 79]. This simplification does not affect the imposed deformation because the loading is prescribed from the geometric difference between the healthy and iNPH ventricles, which represents the net anatomical outcome of all contributing mechanisms. The tissue properties used were from mechanical testing of parenchyma containing interstitial fluid [57], so the contribution of the interstitial fluid to the bulk response is represented in a homogenised sense. Representing these fluid mechanisms explicitly would require a poroelastic or biphasic formulation together with iNPH‐specific fluid transport data. Similarly, blood flow and pressure pulsations associated with cardiac and respiratory cycles, and disease‐driven changes in the mechanical properties of periventricular tissue, are not represented in the quasi‐static formulation used here, and would require a time‐resolved formulation and experimental characterization of iNPH‐affected tissue, respectively. Despite these simplifications, the strong agreement between model predictions and established radiological and morphological markers of iNPH supports our modeling choices for addressing the research questions. Future work comparing hyperelastic, poroelastic and biphasic formulations within the same framework, and extending the model to include fluid dynamics and pulsatile loading, would allow the relative contributions of solid and fluid mechanics to iNPH progression and pathology to be separated.

In addition to the material formulation, the model also relies on group‐averaged MRI data to represent the healthy baseline and the ventricular loading. We used MRI templates of healthy controls and iNPH patients to develop the brain model and prescribe ventricular enlargement for the model. While the model robustly predicts common iNPH morphological changes and identifies the tracts under greatest mechanical loading (the corona radiata, corpus callosum and anterior thalamic radiations, all with large effect sizes and all consistently reported as abnormal in iNPH), it cannot capture differences between patients. For instance, although the reduction in interhemispheric distance and narrowing of sulci at the high convexity have been reported in the literature as a common feature of iNPH [22, 28, 29], these features may not exist in MRI scans of all iNPH patients. This can be related to the variations in ventricular enlargement or brain anatomy across patients. This variability highlights the need for patient‐specific models, which would incorporate individualized neuroanatomy and ventricular loading. Applying the FE‐based registration to all 22 patients in this cohort reproduced individual ventricular anatomy to a median HD95 of 1.12 mm, comparable to the accuracy obtained for the iNPH template. However, the absence of baseline (pre‐disease) MRI scans for iNPH patients currently prevents individualized strain prediction. Our displacement‐based loading relies on computing the geometric difference between a healthy starting state and a diseased state. Applying a generalized healthy template to a specific iNPH patient's unique anatomy would introduce geometric mismatches, for example, discrepancies in overall head size and individual sulcal topology, leading to artificial and unreliable strain predictions. Inter‐patient variability in these anatomical features may alter the magnitude of strain across individual patients. Overcoming this barrier to patient‐specific modeling may be possible by using techniques, such as machine learning generative models, which have been successfully applied to cardiac modeling [80], to estimate a patient's pre‐disease brain geometry. Such approaches require large healthy‐population cohorts to learn the underlying anatomical distribution, and their applicability to iNPH remains to be established. Another barrier against developing patient‐specific models is the highly time‐consuming FE‐based registration, which was used to determine the displacement field of ventricular linings, achieving a successful registration with HD95 of 2.2 mm between the templates and a median of 1.12 mm across individual patients. Each of those registrations is a separate finite element simulation with its related tunings and refinements, so the registration cost scales with the number of patients. A promising replacement for this approach is a machine learning surrogate that maps the healthy and iNPH ventricular geometries directly to the nodal displacement field, bypassing iterative registration. Graph neural networks are a natural fit for this task, since the FE mesh is intrinsically a graph, and such surrogates have been shown to reduce brain deformation simulation time by orders of magnitude without compromising accuracy [81]. Progress in both directions would bring the digital model described in this study a step closer to a true digital twin of an iNPH patient.

A limitation of the present study is that a direct correlation between predicted strain values and individual clinical scale scores was not established. This is because the current model produces group‐level (template‐based) strain predictions rather than patient‐specific strain maps, as discussed above. Achieving such correlations would require patient‐specific strain prediction, which is currently limited by the absence of pre‐disease baseline imaging. Imaging acquired before and after shunt surgery in the same patient would partially address this constraint, since both the initial and the deformed states are then available for that individual. Extending this framework to also incorporate pre‐ and post‐shunt strain reductions alongside clinical outcome data would provide the basis for an integrated predictive tool for patient stratification and shunt‐response prediction. Such a tool would combine, for each patient, the radiological markers already used in clinical assessment with tract‐level strain and its change following shunting, to predict outcome measures such as change in symptom scores or shunt responder status.

In conclusion, this study introduces a novel finite element model of iNPH that leverages detailed brain anatomy and a displacement‐based loading approach to reproduce ventricular expansion with high accuracy. The model is validated by accurate prediction of key morphological changes and tissue abnormalities observed in iNPH, supporting the role of mechanical loading in iNPH pathophysiology. This computational model can be used to investigate the mechanical effects of ventricular enlargement on white matter tracts. Future studies should apply the model to the brain of iNPH patients who underwent CSF drainage to assess the reversibility of strain caused by hydrocephalus and the associated brain abnormalities. This holds promise in patient stratification and for informing shunting procedures.

## 4 Methods

### 4.1 Brain MRI Templates

Standard brain templates, which are average MRI images of healthy individuals, are commonly used for computational model development [82]. However, these templates are for healthy young adults, whereas iNPH predominantly affects elderly individuals aged 60 and above. To represent the brain anatomy of healthy elderly adults and conduct the study based on the pipeline illustrated in Figure 10, we created a template by averaging T1‐weighted MRI scans of 30 healthy individuals aged 62–82 years (16 males, mean age = 73.80 ± 5.71) using antsMultivariateTemplateConstruction.sh from the ANTs library (Figure 10a) [83]. An initial average template was generated by registering all subjects to one reference subject, and this average was then registered to the MNI152 atlas affinely. All scans were subsequently realigned to the evolving template using rigid registration with three iterations to improve accuracy. This process was then repeated with affine and finally symmetric diffeomorphic registration to further refine the averaging. To represent iNPH brains, another template was created using MRI scans of 22 iNPH patients (14 males, mean age = 71.91 ± 7.06) following the same procedure [84].

**FIGURE 10:**
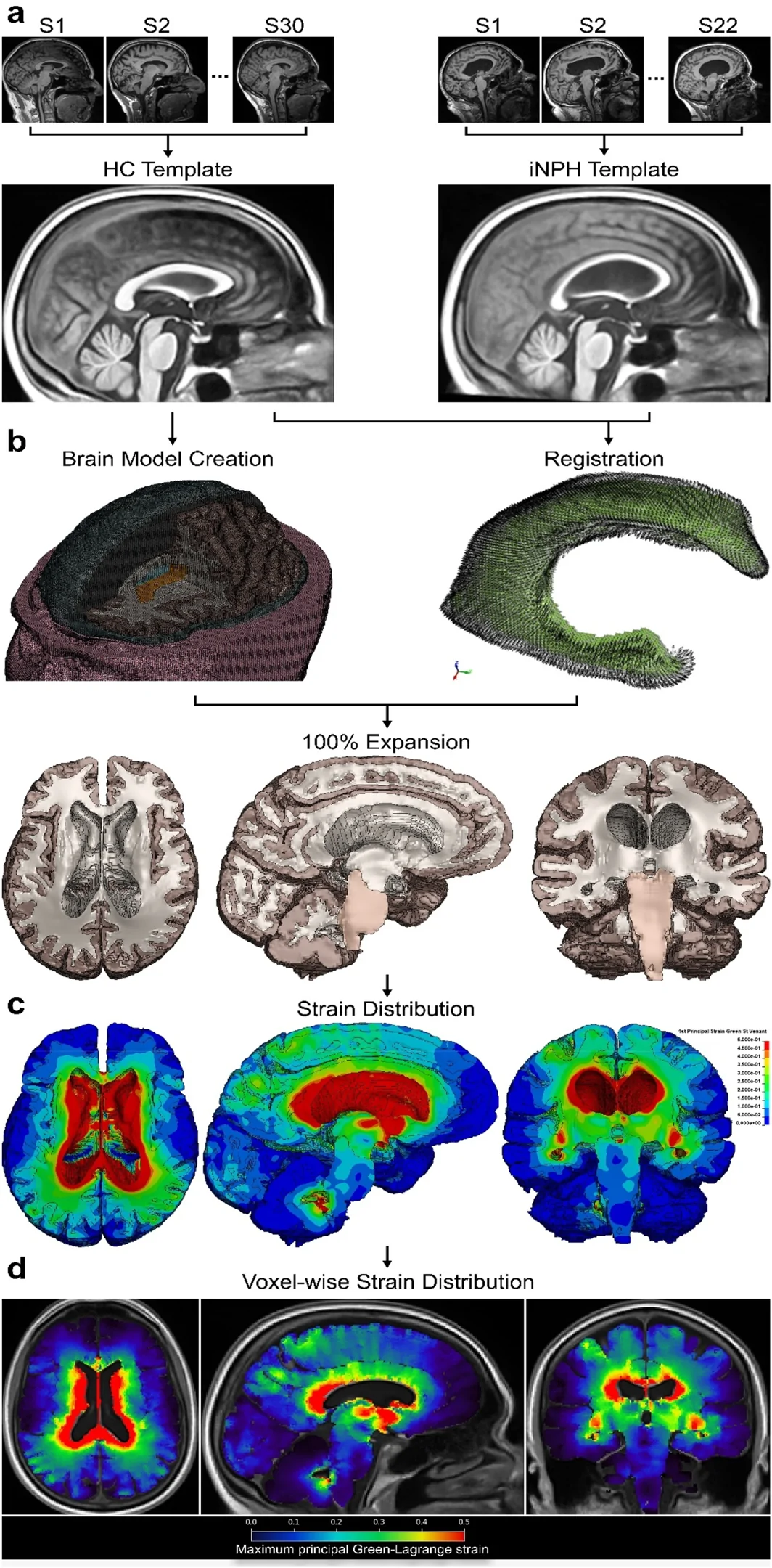
iNPH brain model pipeline. (a) MRI scans of 30 healthy older adults and 22 iNPH patients were used to generate healthy control (HC) and iNPH templates, respectively. (b) Anatomically detailed brain finite element model generated using the HC template. Registration of HC to iNPH ventricles was used to define a 3D displacement field for lining of ventricles, (c) Strain distribution shown on the deformed brain, resulting from the enlargement of the ventricles, (d) Strain distribution was written in NIfTI MRI format for subsequent analysis, such as extracting strain in white matter tracts.

All individuals participating in this study, including iNPH patients and healthy controls, were scanned at the Imperial College Clinical Imaging Facility using a 3 T Siemens Magnetom Verio Syngo with a 32‐channel head coil. A T1‐weighted MPRAGE sequence was used with a duration of 5.03 min using the following parameters: FlipAngle 9°, TR 2300 ms, TE 2.98 ms, TI 900 ms, bandwidth 240 Hz/pixel, acquisition matrix 256 × 240 × 160, voxel size 1.0 × 1.0 × 1.0 mm. All iNPH patients had an existing clinical diagnosis and their cases were reviewed as part of this study by a multidisciplinary team of neurologists, neuroradiologists and psychiatrists to confirm their diagnosis.

### 4.2 Image Segmentation

The HC template was segmented using FMRIB and Freesurfer software [85, 86] for mesh creation. The quality of the segmentation was then checked to ensure the accuracy. This segmentation allows preservation of anatomical features such as sulci and ventricles, which are key for brain biomechanical analyses [41]. Although the importance of sulci has been demonstrated in a 2D computational model of iNPH [52], no prior 3D FE models of iNPH have included sulci. Here, we incorporated sulci in the model to explore their deformation during ventricular expansion. Automatic segmentation of the ventricles can be inaccurate. Hence, all four ventricles were manually segmented in both the HC and iNPH templates using 3D Slicer [87]. This segmentation included the lower parts of the lateral ventricles and the 3rd and 4th ventricles, which are missing in previous models of iNPH [47, 49, 50, 51, 52].

### 4.3 Brain Finite Element Mesh

The segmented HC template was converted into finite element (FE) mesh using a custom in‐house code developed at the HEAD Lab (Figure 10b) [44, 56]. This was achieved by creating a hexahedral element at the location of each brain voxel. This approach results in sharp element edges at the surfaces and interfaces, which can cause simulation instability and stress concentration. Hence, a mesh smoothing algorithm was applied to the mesh. The final brain mesh (excluding CSF, ventricles, skin, and skull) was composed of 1,058,711 elements, with an average element size of 0.991 mm, minimum characteristic length of 0.262 mm, and maximum characteristic length of 1.374 mm. Mesh quality was assessed using PyVista and confirmed to meet accepted quality thresholds for aspect ratio and scaled Jacobian (Supporting Information S1: Figure S1).

### 4.4 Other Brain Structures Incorporated in the Model

Septum Pellucidum: The SP separates the two lateral ventricles, is comprised of both gray and white matter, and is connected to the bottom of the corpus callosum. It typically measures 1–3 mm in thickness [88] and has been implicated in neurological disorders [63]. Despite its significance, no prior iNPH models included a definition of SP. To enhance the biofidelity of our model, a layer of hexahedral elements with an average thickness of 2 mm was added to the mesh using the segmentation refinement pipeline described earlier.

Falx: The falx separates the left and right hemispheres and plays a critical role in the brain's biomechanical behavior [89, 90]. In this study, the falx was modeled as a shell with a thickness of 0.91 mm [91]. Contact algorithms were implemented to capture interactions between the falx and the corpus callosum as well as between the falx and the left and right brain hemispheres.

Pia mater: The pia mater, a thin membrane surrounding the brain, was modeled as a shell with a thickness of 0.2 mm. This layer is essential for maintaining stability at the brain's outer surface and contributes to overall model accuracy, particularly by its interactions with the inner skull.

### 4.5 Material Model of Brain Tissue and Pia Mater

Hyperelastic models, particularly the isotropic Ogden model, have been used to model the stress‐strain behavior of brain tissue under various loading conditions such as compression, stretching, and shearing [76]. Similar to a previous model of iNPH [46], we used the Ogden hyperelastic material model, represented by [92]:

(1) $$W=\sum_{i=1}^{3}\sum_{j=1}^{n}\frac{\mu_{j}}{\alpha_{j}}\left(\lambda_{i}^{\alpha_{j}}-1\right)+K\left(J-1-\ln\;J\right).$$

W, $\lambda_{i}^{\alpha_{j}}$, $\mu_{j}$, $\alpha_{j}$, *K* and *J* are potential function, principal stretches, relaxed shear modulus, material coefficient, bulk modulus and volume ratio respectively. The number of terms, n, can range from 1 to 8, and for this study, we used n=2. The constants used in this model are provided in detail in Tables 2 and 3 [41].

**TABLE 2:** Material properties of brain tissue.

Tissue	Density [kgm3]	$\mu_{1}\;\left[\text{Pa}\right]$	$\alpha_{1}$	$\mu_{2}\;\left[\text{Pa}\right]$	$\alpha_{2}$	Poisson's ratio
Brain	1040	53.8	10.1	−120.4	−12.9	0.4994
Brain stem	1040	15.8	28.1	−106.8	−29.5	0.4994

**TABLE 3:** Material properties of the head tissues.

Tissue	Density [kgm3]	$\mu_{1}\;\left[\text{kPa}\right]$	$\alpha_{1}$	Poisson's ratio
Falx and tentorium	1130	25.5	32.9	0.45
Pia mater	1130	2.6	32.9	0.45
	$\tau_{i}\;\left[\text{ms}\right]$	$\tau_{1}=5$	$\tau_{2}=44$	$\tau_{3}=474$
	$G_{i}\;\left[\text{kPa}\right]$	$G_{1}=32$	$G_{2}=29$	$G_{3}=16$

Brain tissue was modeled using a purely hyperelastic Ogden material, explicitly excluding viscoelastic effects. The months‐to‐year timeline of iNPH progression means negligible viscous effects and that deformation is governed entirely by the hyperelastic equilibrium. Furthermore, because explicit computational simulation of this prolonged loading requires drastically compressing the physical timeframe (as detailed in the subsequent section), including viscous terms would inadvertently introduce artificial, rate‐dependent stiffening. We modeled the pia mater using a model that combines hyperelastic and viscous effects, similar to previous studies that have used visco‐hyperelastic models to simulate quasi‐static problems [93]. This approach prevented numerical instabilities caused by the contact between the pia and skull.

### 4.6 Loading and Boundary Conditions

We loaded the brain model by expanding the ventricles. In previous FE models of iNPH, ventricular enlargement was simulated by applying pressure on the boundaries of the lateral ventricles. However, this approach cannot accurately reproduce the shape of enlarged ventricles [25]. Here, we apply a three‐dimensional displacement field on the lining of ventricles. The displacement field is determined by registering the ventricles of the HC template with the iNPH template. We used two different approaches to achieve the most accurate alignment: (i) linear and nonlinear image registration (image‐based registration), and (ii) finite element biomechanical modeling registration (FE‐based registration).

#### 4.6.1 Image‐Based Registration

For the image‐based registration, we selected two widely used neuroimaging registration techniques that represent the two principal classes of deformation models: linear (affine) and nonlinear (diffeomorphic) registration. Linear registration was performed using FSL FLIRT, which estimates a global affine transformation based on uniform scaling, rotation and shear between two MRI images. The resulting transformation matrix was applied directly to the HC mesh to generate an approximation of the iNPH ventricle. Nonlinear registration was also performed using the ANT SyN algorithm, a diffeomorphic approach that models spatially varying, nonuniform deformations. Unlike affine registration, SyN allows each region of the ventricle to deform independently, enabling the algorithm to better accommodate complex geometric changes than FSL FLIRT. This algorithm included an initial affine registration followed by deformable warping. As this method generates a dense voxel‐wise displacement field rather than a global transformation matrix, the output could not be applied directly to the HC mesh in the same manner as the linear approach. As a result, the non‐linearly registered image of the HC ventricle was directly converted into a mesh to represent the iNPH ventricle instead of extracting any registration matrix or displacement field from the registration process.

#### 4.6.2 FE‐Based Registration

FE meshes of the brain were generated from the HC and iNPH templates. The solid elements of the ventricles were extracted from the brain meshes. Then, the shell elements were generated on the surface of the ventricles, and the internal solid elements were removed. This process produced paired shell meshes for each ventricle representing the HC and iNPH ventricles.

Next, the shell mesh of the HC ventricle was expanded by applying unit pressure to each element until it reached the fixed iNPH surface. An automatic surface to surface contact algorithm with a soft constraint penalty formulation (scale factor of 0.1) was defined between the two surfaces to prevent interpenetration during expansion simulation. Parameters including material properties and shell thickness were iteratively tuned to maximize numerical stability and achieve optimal geometric registration, rather than to represent physiological tissue properties. Successful registration was achieved using an elastic material model (ρ=1000[kgm3], elastic modulus = 10 [kPa], Poisson's ratio = 0.3) and Belytschko‐Tsay membrane element formulation for both meshes. Shell thicknesses were set to 3 mm for the HC and 0.1 mm for the iNPH mesh. The process of FE‐based registration is illustrated in Figure 11.

**FIGURE 11:**
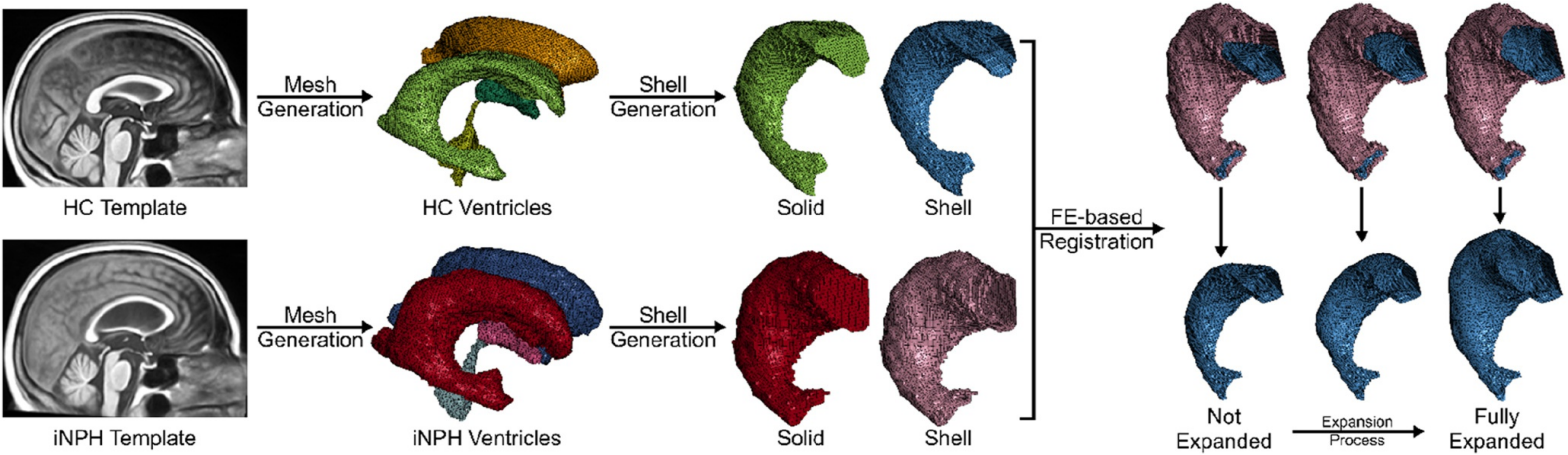
FE‐based registration pipeline. iNPH and HC ventricular meshes were generated from templates using an in‐house pipeline. Shell surfaces were created for the right ventricles of each group. During registration, the iNPH ventricle was fixed, and the HC ventricle was expanded to conform to the iNPH surface. This registration process was subsequently repeated for other sets of ventricles.

The difference between the positions of the HC ventricle nodes before and after expansion was calculated. This resulted in a three‐dimensional displacement field for the solid element nodes located on the surface of the HC ventricles.

The procedure was repeated independently for both lateral, third and fourth ventricles, resulting in a complete set of nodal displacements describing the transformation from HC to iNPH geometry. The expansion simulations were performed using LS‐DYNA, and the displacement vectors were extracted via a custom Python script.

#### 4.6.3 Quantifying Registration Accuracy

Registration accuracy was quantified by using 95th percentile Hausdorff distance (HD95) [59]. While the standard Hausdorff distance is sensitive to noise, the 95th percentile variant is widely recommended for medical imaging as it mitigates the impact of outliers [94]. To compute the HD95, the distance from each node on the registered HC ventricular mesh to the nearest point on the reference iNPH ventricular surface was calculated. The HD95 was defined as the distance value below which 95% of these nodal deviations fall. This approach provides a reliable assessment of the spatial correspondence between the registered HC and reference iNPH ventricular geometries, effectively characterizing surface mismatch.

### 4.7 Controlling Kinetic and Hourglass Energies

We used the nonlinear explicit solver of LS‐Dyna to predict brain deformation caused by ventricular enlargement. Explicit solvers can simulate highly nonlinear problems, such as ventricular enlargement, which involves geometric nonlinearity, material nonlinearity and contact. iNPH develops over months to years, but it is not feasible to run LS‐Dyna's explicit solver for such long durations. However, it is possible to reduce the loading duration by orders of magnitude as long as the dynamic effects remain negligible. A loading duration of 500 milliseconds was used, selected to preserve quasi‐static behavior (kinetic energy below 5% of internal energy) while remaining computationally feasible. A sensitivity analysis across a broader range of loading durations confirmed that the predicted tract‐level strain is invariant to loading duration within this regime (Supporting Information S1: Figure S2).

To avoid numerical instabilities arising from large shearing deformations and nearly incompressible properties of the brain, we used reduced integration for solid elements of the brain. When reduced order integration is used, spurious deformations, often called hourglass deformation modes, can occur. These deformations should be controlled via using the hourglass control. A stiffness‐based hourglass control was applied and tuned to ensure that the hourglass energy remained below 10% of the internal energy while preserving numerical stability throughout the simulation (Supporting Information S1: Figure S3).

### 4.8 Tract‐Based Strain Distribution

After completion of the ventricular expansion simulation, Green–Lagrange strain was calculated for each element across the brain based on nodal displacements (Figure 10c). These element‐wise strains were converted to voxel‐wise strain maps and written in NIfTI MRI format using an in‐house pipeline, allowing the biomechanical outputs to be analyzed using standard neuroimaging tools (Figure 10d). To enable tract‐based analysis, the JHU ICBM‐DTI‐81 white matter tract atlas was used to define anatomical regions for sampling the strain values [64].

It should be noted that, as the JHU ICBM‐DTI‐81 white matter tract atlas is provided in the MNI152 space (Figure 12a,b), it could not be directly applied to the HC template space. To address this, the MNI152 template was nonlinearly registered to the HC template using ANTs. The resulting deformation field was applied to the tract masks, bringing them into the HC space (Figure 12c,d). These transformed tract masks were then overlaid onto the voxel‐wise strain maps, and voxel values within each mask were extracted. For each tract, strain distributions were quantified by computing voxel‐wise strain values within the corresponding mask, enabling identification of tract‐specific deformation patterns associated with ventricular enlargement.

**FIGURE 12:**
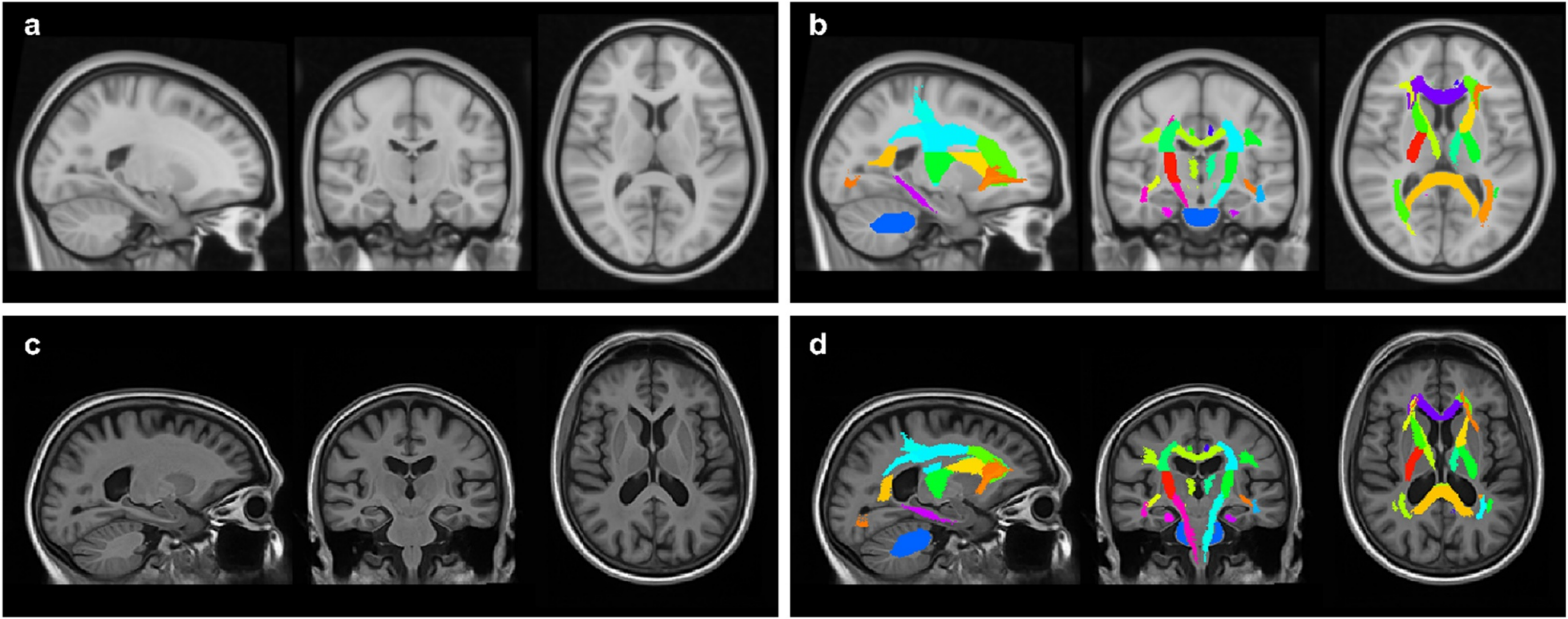
Process of transforming the JHU ICBM‐DTI‐81 white matter tract atlas into HC space. (a) MNI152 template. (b) JHU ICBM‐DTI‐81 white matter tract atlas in MNI152 space. (c) HC template. (d) white matter tract atlas transformed into HC space.

### 4.9 Patient‐Specific FE‐Based Registration

To assess the applicability of the presented framework for patient‐specific modeling, the FE‐based registration was repeated with each patient's own ventricles as the target, replacing the iNPH template. The T1‐weighted scan of each patient was rigidly registered into the HC template space using the ANTs library, and the lateral ventricles were segmented and converted into a shell surface using the same method described for the iNPH template. In each patient‐specific registration, the patient's ventricular surface was used as the fixed target of the expansion.

The HC ventricular shell mesh was used unchanged as the expanding surface. The same as the template registration, material properties, shell thickness and expansion duration were tuned to maximize numerical stability and geometric registration accuracy rather than to represent physiological tissue properties. Registration accuracy was quantified using the HD95, computed between the nodes of the expanded HC mesh and the surface of the patient's segmented ventricle, as described above. The expansion simulations were performed using LS‐DYNA and the registered geometries were analyzed with custom Python scripts.

## Author Contributions

M.G., D.J.S., C.C., and P.A.M. conceived and designed the study. V.D. developed the brain model. V.D. and M.G. designed the method for extracting the new loading conditions. V.D. validated the model, analyzed the predictions against radiological data, and conducted the final strain prediction analysis. M.D.G., M.C.B.D., and M.A.K. collected and processed the MRI scans. V.D. and M.D.G. performed registrations. B.K.D. and V.D. performed patient‐specific analysis. A.G. calculated the radiological scores. M.G., D.J.S., C.C., and P.A.M. supervised the study. V.D., M.G., and M.D.G. wrote the manuscript. All authors reviewed the data and approved the submission.

## Ethics Statement

The acquisition of MRI data for this study was approved by the Health Research Authority's London‐Surrey Borders Research Ethics Committee (19/LO/0102), the London‐Central Research Ethics Committee (18/LO/0249), the Camberwell St Giles Research Ethics Committee (17/LO/2066), and the Imperial College Research Ethics Committee (17IC4009).

## Consent

All participants provided digital or written informed consent.

## Conflicts of Interest

D.J.S. serves on the Alzheimer’s Society Research Advisory Board and the Rugby Football Union Concussion Advisory Board. D.J.S. serves on the Editorial Board of the journal *Brain*. D.J.S. has received research contributions from Alamar, although unrelated to this work. C.C. has received honoraria for B‐Braun (lectures) and Roche (advisory board). P.A.M. reports funding from the National Institute for Health and Care Research (NIHR), Alzheimer’s Research UK (ARUK), Federation Internationale de Football Association (FIFA), the Football Association (FA), LifeArc, Dementias Platform UK, the Medical Research Council (MRC), and the UK Dementia Research Institute (UK DRI). He also participates in an independent Data Safety Monitoring Board for Johnson & Johnson, is a Trustee of the Alzheimer’s Society, and is the NIHR Research Delivery Network (RDN) National Specialty Lead for Dementia and Neurodegeneration. The other authors declare no conflicts of interest.

## Code Availability

The finite element simulations were performed using the commercial software LS‐DYNA (Version R15, Ansys). Custom code for generating displacement fields and data analysis was implemented in Python (version 3.11.9). All custom code is available from the corresponding author upon reasonable request.

## Supporting information


Supporting Information S1

## Data Availability

The data that support the findings of this study are not publicly available due to privacy and ethical restrictions. They are available from the corresponding author upon reasonable request and following the completion of a material transfer agreement with Imperial College London.
