# Supplementary material for: Patient‐Informed Biomechanical Modeling Reveals Mechanical Mechanism of Brain Damage in Idiopathic Normal Pressure Hydrocephalus
Source: Smart Med. 2026 Oct 30;5(5):e70060. doi: 10.1002/smmd.70060 (PMC13720981; doi:10.1002/smmd.70060)
Supplement: Supplementary file 1 [file SMMD-5-e70060-s001.docx]

# Supporting Information

***Mesh quality assessment***

Mesh quality assessment was conducted using the PyVista library in Python. Results indicated that 98.5% of elements exhibited a good aspect ratio (< 3), 95.2% had an acceptable Jacobian ratio (< 10), and 81.2% maintained an acceptable scaled Jacobian (> 0.5), as illustrated in FIGURE S1.

**FIGURE S1** | **Mesh quality assessment of the brain FE model.**

***Loading duration and energy monitoring***

To identify an optimal balance between negligible dynamic effects and loading duration, expansion durations ranging from 25 to 1000 milliseconds were simulated, corresponding to run times of approximately 40 minutes to 19 hours, respectively. Internal and kinetic energy of the brain were monitored. As a rule of thumb, when kinetic energy remained below 5% of the internal energy, we assumed dynamic effects were negligible, preserving the characteristics of iNPH progression. The results indicated that using 500 milliseconds as the overall expansion time allowed for a faithful representation of the disorder while maintaining computational feasibility.

To confirm that this choice does not influence the reported strain results, a sensitivity analysis was performed in which the full simulation and the complete tract-based strain pipeline were repeated at expansion durations of 25, 50, 100, 250, 500, 750 and 1000 milliseconds (FIGURE S2). The 1000 millisecond simulation was taken as the reference. The kinetic-to-internal energy ratio decreased monotonically with duration and fell below the 5% criterion from 50 milliseconds onward, reaching 0.27% at 500 milliseconds (FIGURE S2a). Tract-level convergence, however, required considerably longer durations. At 50 milliseconds the whole-brain 95^th^ percentile strain deviated from the reference by only 0.37% and the median tract by 0.52%, yet the most-affected tract still deviated by 7.2% and 9 of the 32 tracts remained outside 2% (FIGURE S2c AND FIGURE s2d). All tracts fell within 5% of the reference only for durations of 250 milliseconds or longer. At the 500 millisecond duration used throughout this study, the largest deviation of any tract was 2.2% and the whole-brain deviation was 0.17%, confirming that the reported tract strains are independent of the expansion duration.

**FIGURE S2** | **Independence of tract-level strain predictions from the duration of ventricular expansion. a,** Ratio of kinetic to internal energy at the end of each simulation, which falls below the 5% quasi-static limit (dashed line) from 50 ms onward. **b**, Percentage of the 32 white matter tracts whose 95^th^-percentile strain lies within 2% and 5% of the reference. **c**, Deviation of the 95^th^-percentile maximum principal Green–Lagrange strain from the reference, shown for every tract (orange points), the tract median, the most-deviating tract, and the whole brain. **d**, Magnified view of the shaded region in c. The amber band in a–d marks the durations over which the kinetic-to-internal energy ratio and the whole-brain strain have both converged while the most-deviating tract has not.

***Hourglass control***

We applied a stiffness-based hourglass control, with coefficients ranging from 0.01 to 0.5. The hourglass energy, which is an artificial energy produced by the hourglass control, was monitored to ensure it remains less than 10% of the internal energy. When the hourglass coefficient was small, the simulation crashed due to numerical instability (FIGURE S3). Increasing the hourglass coefficient improved numerical stability, allowing the simulation to progress further. We selected a coefficient that allowed for the simulation of a fully enlarged ventricle while ensuring the hourglass energy is small enough.

**FIGURE S3** | **Effect of hourglass settings on simulation accuracy and model stability.**

***Patient-specific FE-based registration***

The FE-based registration was applied to the ventricles of every patient in the cohort, as described in the Methods and reported in the Results. FIGURE S4 shows the outcome of every registration, with the corresponding HD95.

**FIGURE S4** | **FE-based registration of the HC ventricle onto the ventricles of each patient.** Each panel shows the patient’s segmented ventricle (red) and the registered HC mesh (yellow), with the HD95 for that registration given in the panel. Each row is one patient.
